# Comparison of dentoskeletal and soft tissue effects of Class II malocclusion treatment with Jones Jig appliance and with maxillary first premolar extractions

**DOI:** 10.1590/2177-6709.24.2.056-065.oar

**Published:** 2019

**Authors:** Daniela Cubas Pupulim, José Fernando Castanha Henriques, Guilherme Janson, Fernanda Pinelli Henriques, Karina Maria Salvatore Freitas, Daniela Garib

**Affiliations:** 1 Universidade de São Paulo, Faculdade de Odontologia de Bauru, Departamento de Ortodontia (Bauru/SP, Brazil).; 2 Centro Universitário Ingá, Departamento de Ortodontia (Maringá/PR, Brazil).

**Keywords:** Malocclusion, Angle Class II, Orthodontic appliances, Tooth extraction

## Abstract

**Objective:**

The aim of this study was to compare the cephalometric changes in Class II division 1 malocclusion patients treated with Jones Jig appliance or with maxillary first premolar extractions.

**Methods:**

The sample consisted of 88 lateral cephalograms of 44 patients, divided into two groups. Group 1 consisted of 21 patients treated with Jones Jig appliance, with a mean initial age of 12.88 ± 1.23 years and final mean age of 17.18 ± 1.37 years, and a mean treatment time of 4.29 years. Group 2 comprised 23 patients treated with maxillary first premolar extractions, with a mean initial age of 13.59 ± 1.91 years and mean final age of 16.39 ± 1.97 years, and a mean treatment time of 2.8 years. Intergroup treatment changes were compared with *t* and Mann-Whitney tests.

**Results:**

Class II correction in G2 (maxillary first premolar extractions) presented significantly greater maxillary retrusion, reduction of anteroposterior apical base discrepancy, smaller increase in the lower anterior face height and significantly greater overjet reduction than G1 (Jones Jig).

**Conclusions:**

Treatment with maxillary first premolar extractions produced greater overjet reduction, but the two treatment protocols produced similar changes in the soft tissue profile.

## Resumo

**Objetivo:**

o objetivo desse estudo foi comparar as alterações cefalométricas em pacientes com má oclusão de Classe II, divisão 1, tratados com o aparelho Jones Jig ou com extrações dos primeiros pré-molares superiores.

**Métodos:**

a amostra consistiu de 88 telerradiografias laterais de 44 pacientes, que foram divididos em dois grupos. O Grupo 1 consistiu de 21 pacientes tratados com o aparelho Jones Jig, com idade inicial média de 12,88 ± 1,23 anos e idade final média de 17,18 ± 1,37 anos, com tempo médio de tratamento de 4,29 anos. O Grupo 2 compreendeu 23 pacientes tratados com extrações dos primeiros pré-molares superiores, com idade inicial média de 13,59 ± 1,91 anos, idade final média de 16,39 ± 1,97 anos, e tempo médio de tratamento de 2,8 anos. A comparação intergrupos das alterações dos tratamentos foi realizada pelos testes *t* e de Mann-Whitney.

**Resultados:**

a correção da Classe II no G2 (extrações dos primeiros pré-molares superiores) apresentou uma retrusão maxilar significativamente maior, redução da discrepância anteroposterior das bases apicais, menor aumento da altura facial anteroinferior e uma redução significativamente maior do trespasse horizontal do que no G1 (aparelho Jones Jig).

**Conclusões:**

o tratamento com extrações dos primeiros pré-molares superiores promoveu maior redução do trespasse horizontal, mas os dois protocolos de tratamento produziram alterações semelhantes no perfil tegumentar.

## INTRODUCTION

Class II malocclusion correction can be achieved by means of various orthodontic mechanics. The choice of the most appropriate treatment plan should take into consideration the initial malocclusion severity, patient age, growth pattern, soft tissue profile, patient compliance and the patient’s chief complaint.

In non-extraction treatment of dental Class II malocclusion, the headgear is usually used to distalize maxillary molars to a Class I molar relationship. However, the lack of patient compliance can reduce treatment effectiveness.^1^ Thus noncompliance intraoral distalizing appliances were developed to simplify distalization of maxillary molars and especially as an alternative for noncompliant patients.^2^ ^,^ ^3^

Among noncompliance intraoral distalizing appliances to correct Class II malocclusions, the Jones Jig appliance presents as a good option because it is easy to install, and provides a fast and efficient correction in the molar relationship.^4^

Treatment of Class II malocclusions with maxillary first premolar extractions would be recommended in cases with moderate skeletal discrepancies,^5^ reduced amount of mandibular crowding,^6^ ^-^ ^8^ protrusion and/or crowding of the maxillary incisors,^8^ horizontal growth pattern^8^ and variable amount of overbite to achieve a harmonious facial profile.^5^ In this treatment protocol, the objective is to finalize the molars in a Class II relationship, Class I canine relationship, with normal overjet and overbite.

Several studies have evaluated the dentoskeletal and soft tissue effects produced by the Jones Jig appliance,^4^ ^,^ ^9^ ^,^ ^10^ while others have evaluated the effects produced by maxillary first premolar extractions in Class II correction.^1^ ^,^ ^7^ ^,^ ^11^ ^-^ ^15^ However, there are no studies comparing the cephalometric dentoskeletal and soft tissue effects of these two treatment protocols.

Therefore, the aim of this study was to compare the dentoskeletal and soft tissue effects of patients with Class II malocclusion treated with Jones Jig appliance and with maxillary first premolars extractions.

## MATERIAL AND METHODS

Ethical approval of this retrospective study was obtained from the Bauru Dental School, University of São Paulo, and parents of the patients signed an informed consent before inclusion in the study. 

Sample size calculation was performed based on an alpha level of significance of 5% and beta of 20%, to achieve a power of 80% of the test, to detect a mean difference of 1.25 ± 1.4 mm in overjet change.^15^ The calculation showed that 21 patients were needed in each group.

In this retrospective clinical study, 88 lateral cephalograms of 44 patients with Class II malocclusion from the files of Bauru Dental School, University of São Paulo were used. Sample selection was based on the following criteria: patients who initially presented with bilateral Class II malocclusion and who were treated with the Jones Jig appliance or with maxillary first premolars extractions and fixed edgewise appliances; Class II malocclusion with minimum anteroposterior severity of ¼ Class II molar relationship as evaluated on the study models; presence of all permanent teeth up to the first molars; mild to moderate crowding in the maxillary arch; no previous orthodontic treatment; and with complete orthodontic records.

Lateral cephalograms of each patient were obtained before and after treatment. The sample was divided into two groups. Group 1 consisted of 21 patients (11 male, 10 female), with a mean initial age of 12.88 years (SD = 1.23; range = 11.65 - 14.11 years) who were treated with the Jones Jig and fixed appliances during a mean time of 4.29 years (SD = 0.76; range = 3.53 - 5.05 years). Eight patients had one quarter-cusp Class II molar relationship, eight had one half-cusp Class II molar relationship and five had three-quarter Class II molar relationship.^16^ Molar relationship was corrected with the Jones Jig appliance, as described by Patel et al.^17^ The original stainless steel coil spring was changed to a Nitinol coil spring (G&H Wire Co, Greenwood, Ind) to apply continuous force. The coil spring was activated 5 mm every four weeks, to deliver 120 grams (0.12N) of force, in average. A Nance button was also used as anchorage in the maxillary second premolars.^2^ ^,^ ^10^ The Jones Jig appliance was used until the maxillary first molars were distalized to a Class I relationship. The mean molar distalization time was 0.80 years (SD = 0.20; range = 0.60 - 1.00 year). Then, 0.022 x 0.028-inch fixed orthodontic appliances (Roth prescription) were installed. Leveling and alignment followed the usual wire sequence characterized by an initial 0.014-in or 0.016-in nitinol, followed by 0.016, 0.018, 0.020, and 0.018 x 0.025-in stainless steel archwires. Deep overbite was corrected with accentuated and reversed curves of Spee. Sequential retraction of the second premolars followed by the first premolars was performed with elastic chains on the rectangular archwire. During *en masse* anterior retraction, 3/16-in Class II elastics were used 12 to 20 hours/day, releasing an average force of 200g/side. Anchorage reinforcement was provided by a headgear at night, when necessary. Maxillary third molars were not extracted, when present.

Group 2 comprised 23 patients (11 male, 12 female) who had maxillary first premolars removed during their comprehensive orthodontic treatment, for a mean treatment time of 2.80 years (SD = 0.88; range = 1.92 - 3.68 years), with a mean initial age of 13.59 years (SD = 1.91; range = 11.68 - 15.5 years). Four patients had one quarter-cusp Class II molar relationship, sixteen had one half-cusp Class II molar relationship and three had three-quarter Class II molar relationships.^16^ Four patients were initially planned to non-extraction treatment and use of extraoral headgear. However, due to lack of patient compliance in using the headgear, treatment was reversed to maxillary first premolar extractions. Conventional fixed Edgewise appliances, with 0.022 x 0.028-in slot dimensions were used. Leveling and alignment followed the usual wire sequence characterized by an initial 0.014-in or 0.016-in nitinol, followed by 0.016, 0.018, 0.020, and 0.021 x 0.025-in or 0.018 x 0.025-in stainless steel archwires. Deep overbite was corrected with accentuated and reversed curves of Spee. Maxillary *en masse* anterior retraction was performed with elastic chains, from the anterior hook to the hook of the maxillary first molars. In the more accentuated Class II malocclusions, an extraoral headgear and Class II elastics were used to reinforce anchorage, when necessary. Both groups finished with very acceptable occlusions, normal overbite and overjet, Class I canine relationships and no posterior crossbites.^16^

**Figure 1:**
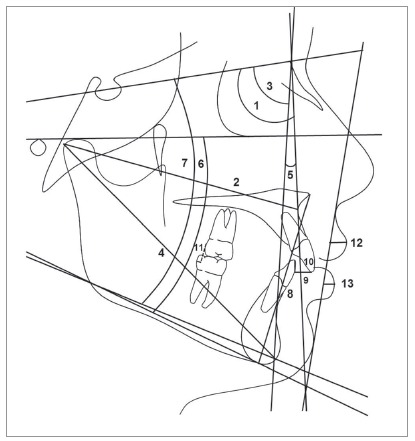
Linear and angular skeletal, dentoalveolar relationships and soft tissue profile measurements: 1) SNA (degrees); 2) Co-A (mm); 3) SNB (degrees); 4) Co-Gn (mm); 5) ANB (degrees); 6) FMA (degrees); 7) SN.GoGn (degrees); 8) LAFH (mm), 9) Overjet (mm); 10) Overbite (mm); 11) Molar relationship (mm); 12) UL-SnPg’ (mm); 13) LL-SnPg’ (mm).

**Figure 2:**
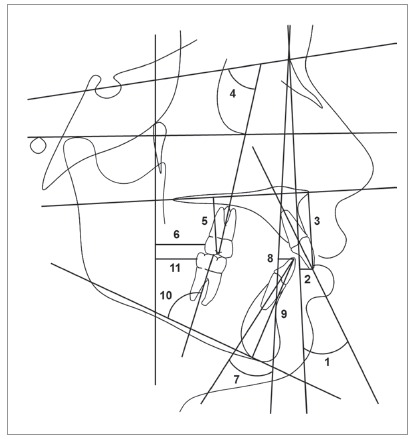
Linear and angular maxillary and mandibular dentoalveolar measurements: 1) Mx1.NA (degrees); 2) Mx1-NA (mm); 3) Mx1-PP (mm); 4) Mx6.SN (degrees); 5) Mx6-PP (mm); 6) Mx6-PTV (mm); 7) Md1.NB (degrees); 8) Md1-NB (mm); 9) Md1-MP (mm); 10) Md6-MP (mm); 11) Md6-PTV (mm).

The pre- (T_1_) and posttreatment (T_2_) cephalometric headfilms were scanned with ScanMaker i800 scanner (Microtek, Hsinchu, Taiwan), with a 300 dpi resolution to allow image acquisition in Dolphin Imaging v. 11.5 software (Dolphin Imaging and Management Solutions, Chatsworth, Calif., USA). This software corrected the magnification factor of the radiographic images, that was between 6% and 9.8%. The cephalometric landmarks and variables used in this study are shown in Figures 1 and 2 and Table 3. 

### Error study

Thirty cefalometric headfilms were randomly selected and remeasured by the same examiner after a 1-month interval. Random errors were calculated according to Dahlberg’s formula:^18^ S^2^ = Σd^2^/2n, where S^2^ is the error variance and ‘d’ is the difference between two determinations of the same variable, and the systematic errors were estimated with dependent *t* tests, at *p*< 0.05.^19^

### Statistical analyses

Shapiro-Wilk tests were used to evaluate data distribution. Some pretreatment and treatment changes variables did not show normal distribution. 

Group comparability regarding initial and final ages and treatment time was evaluated with *t* tests, and sex percentage and severity of Class II malocclusion were evaluated with Chi-square tests.

The pretreatment stage and the treatment changes were compared between the groups. T tests were used for the variables with normal distribution, and Mann-Whitney tests were used for variables without normal distribution.

Because the Jones Jig group (G1) had a significantly greater treatment time, the treatment changes were annualized according to the maxillary first premolar extractions group (G2) treatment time.^20^ ^,^ ^21^ Therefore, all patients in G1 had their individual treatment changes, for each variable, divided by their treatment time, and then multiplied by the mean treatment time of G2.

All statistical analyses were performed with Statistica software (Statistica for Windows, v. 6.0, Statsoft, Tulsa, Okla), and the results were considered significant at *p*< 0.05.

## RESULTS

The random errors did not exceed 1.89 mm (Co-A) or 2.05^o^ (Mx6.SN) and only one variable showed a significant systematic error (Mx6.SN, Table 1).

**Table 1:** Random and systematic errors between the first and second measurements (Dahlberg’s formula and dependent t tests).

Variables	Measurement 1		Measurement 2		Dahlberg	P
	Mean	SD	Mean	SD		
Maxillary component						
SNA (degrees)	81.96	5.19	82.20	5.47	1.16	0.424
Co-A (mm)	83.41	4.84	83.28	4.97	1.89	0.800
Mandibular component						
SNB (degrees)	78.00	4.40	77.91	4.38	0.80	0.681
Co-Gn (mm)	109.68	6.57	109.15	6.36	1.52	0.183
Maxillomandibular relationship						
ANB (degrees)	3.97	1.72	4.29	2.08	0.95	0.199
Vertical component						
FMA (degrees)	25.32	4.39	25.30	4.16	1.24	0.935
Sn.GoGn (degrees)	31.96	4.89	31.94	4.73	0.92	0.924
AFAI (mm)	63.45	6.11	62.80	5.78	1.48	0.090
Maxillary dentoalveolar component						
1.NA (degrees)	23.68	6.58	23.50	6.63	1.25	0.580
1-NA (mm)	4.03	2.02	3.63	2.48	1.24	0.218
1-PP (mm)	27.94	2.58	27.81	2.46	0.92	0.603
6.SN (degrees)	74.61	4.44	73.20	4.73	2.05	0.006*
6-PP (mm)	21.03	2.25	21.02	2.15	0.38	0.948
6-PTV (mm)	16.94	3.90	16.93	3.71	0.93	0.957
Mandibular dentoalveolar component						
1.NB (degrees)	28.33	6.88	28.96	6.82	1.31	0.061
1-NB (mm)	5.31	2.00	5.61	2.12	0.65	0.071
1-MP (mm)	38.59	3.41	38.41	3.42	0.37	0.060
6-MP (mm)	27.72	3.45	27.64	3.72	0.44	0.489
6-PTV (mm)	16.12	4.06	16.00	3.97	1.09	0.669
Dentoalveolar relationships						
Overjet (mm)	4.09	2.04	4.00	1.97	0.24	0.142
Overbite (mm)	2.13	1.68	2.18	1.73	0.20	0.346
Molar relationship (mm)	0.36	1.81	0.31	1.88	0.31	0.539
Soft tissue profile						
UL-SnPg’ (mm)	4.19	1.88	4.23	1.73	0.38	0.663
LL-SnPg’ (mm)	3.89	2.17	4.08	2.19	0.64	0.265

*Statistically significant at *p*< 0.05.

The groups were comparable regarding pre- and posttreatment ages, but treatment time in the Jones Jig group was significantly greater than in the maxillary first premolar extractions group (Table 2). Sex distribution and Class II molar relationship severity were similar in the groups (Table 2).

**Table 2:** Intergroup comparisons of pretreatment and posttreatment ages, and treatment time (t tests), sex distribution (Chi-square test) and severity of Class II molar relationship (Chi-square test).

Variables	Group 1 (n = 21; Jones Jig appliance)		Group 2 (n = 23; maxillary first premolar extractions)		P
	Mean	SD	Mean	SD	
Pretreatment age	12.88	1.23	13.59	1.91	0.153
Post-treatment age	17.18	1.37	16.39	1.97	0.140
Treatment time	4.29	0.76	2.80	0.88	0.000*
Male	11		11		0.762
Female	10		12		
¼ Class II	8		4		0.109
½ Class II	8		16		
– Class II	5		3		

*Statistically significant at *p*< 0.05.

At pretreatment, the maxillary first premolar extractions group (G2) had significantly greater mandibular retrusion, vertical growth pattern, lower anterior face height and overjet than the Jones Jig group (G1), which had significantly greater overbite than G2 (Table 3). During treatment, G2 presented significantly greater maxillary retrusion, reduction of apical bases anteroposterior discrepancy and smaller increase in lower anterior face height than G1 (Table 4).

**Table 3:** Intergroup pretreatment comparison (t test = € and Mann-Whitney test = §).

Variables	Group 1 (n = 21; Jones Jig appliance)		Group 2 (n = 23; maxillary first premolar extractions)		P
	Mean/Median	SD/Interquartile range	Mean/Median	SD/Interquartile range	
Maxillary component					
SNA (degrees)	83.20	4.40	81.34	4.88	0.194^€^
Co-A (mm)	83.54	4.79	82.05	6.27	0.384^€^
Mandibular component					
SNB (degrees)	79.25	3.84	76.58	4.10	0.031^€^*
Co-Gn (mm)	105.95	5.93	104.53	7.10	0.476^€^
Maxillomandibular relationship					
ANB (degrees)	3.93	2.24	4.76	2.05	0.206^€^
Vertical component					
FMA (degrees)	24.79	4.13	27.63	4.19	0.029^€^*
Sn.GoGn (degrees)	32.10	7.00	32.30	6.70	0.102^§^
AFAI (mm)	61.05	4.94	64.65	5.45	0.027^€^*
Maxillary dentoalveolar component					
1.NA (degrees)	24.33	6.09	23.90	6.55	0.819^€^
1-NA (mm)	4.31	2.12	4.56	2.49	0.722^€^
1-PP (mm)	26.27	2.67	27.70	2.61	0.080^€^
6.SN (degrees)	74.10	8.80	72.70	4.20	0.259^§^
6-PP (mm)	20.04	2.30	20.89	2.60	0.260^€^
6-PTV (mm)	15.38	3.87	14.80	3.84	0.625^€^
Mandibular dentoalveolar component					
1.NB (degrees)	26.60	6.40	26.30	5.35	0.864^€^
1-NB (mm)	4.72	2.22	5.06	2.03	0.603^€^
1-MP (mm)	37.52	3.00	38.88	3.76	0.196^€^
6-MP (mm)	26.05	2.70	27.43	3.16	0.128^€^
6-PTV (mm)	14.97	4.52	13.77	4.21	0.368^€^
Dentoalveolar relationships					
Overjet (mm)	4.89	1.73	6.07	2.09	0.048^€^*
Overbite (mm)	3.54	1.30	2.08	2.25	0.013^€^*
Molar relationship (mm)	-0.04	1.38	0.46	1.40	0.226^€^
Soft tissue profile					
UL-SnPg’ (mm)	5.04	1.98	5.28	2.52	0.729^€^
LL-SnPg’ (mm)	4.44	1.81	4.06	2.27	0.539^€^

*Statistically significant at *p*< 0.05.

**Table 4:** Intergroup treatment changes comparison during 2.8 years (T_1_-T_2_, t test = € and Mann-Whitney test = §).

Variables	Group 1 (n = 21; Jones Jig appliance)		Group 2 (n = 23; maxillary first premolar extractions)		P
	Mean/Median	SD/Interquartile range	Mean/Median	SD/Interquartile range	
Maxillary component					
SNA (degrees)	0.25	1.50	-1.21	2.67	0.032^€^*
Co-A (mm)	0.86	1.74	-0.17	3.39	0.216^€^
Mandibular component					
SNB (degrees)	0.64	1.60	-0.60	2.40	0.244^§^
Co-Gn (mm)	3.23	3.33	2.00	6.00	0.341^§^
Maxillomandibular relationship					
ANB (degrees)	-0.26	0.80	-1.51	1.62	0.003^€^*
Vertical component					
FMA (degrees)	0.45	1.73	-0.26	2.53	0.285^€^
Sn.GoGn (degrees)	-0.03	1.26	-0.14	2.15	0.846^€^
AFAI (mm)	3.55	2.35	1.26	3.07	0.008^€^*
Maxillary dentoalveolar component					
1.NA (degrees)	-0.05	5.08	1.20	13.30	0.378^§^
1-NA (mm)	0.11	1.83	0.00	4.90	0.518^§^
1-PP (mm)	0.95	1.37	-0.94	2.07	0.000^€^*
6.SN (degrees)	1.88	2.93	5.61	4.53	0.002^€^*
6-PP (mm)	1.38	1.26	1.40	1.69	0.978^€^
6-PTV (mm)	1.27	1.68	4.58	2.37	0.000^€^*
Mandibular dentoalveolar component					
1.NB (degrees)	2.98	3.20	2.73	6.11	0.863^€^
1-NB (mm)	0.95	0.96	1.06	1.61	0.790^€^
1-MP (mm)	1.44	1.64	1.00	1.32	0.332^€^
6-MP (mm)	2.52	1.35	1.55	1.61	0.037^€^*
6-PTV (mm)	2.48	1.65	1.78	2.79	0.323^€^
Dentoalveolar relationships					
Overjet (mm)	-1.40	1.92	-4.30	2.70	0.000^§^*
Overbite (mm)	-1.17	0.98	-0.70	2.00	0.088^§^
Molar relationship (mm)	-1.30	1.13	2.85	1.31	0.000^€^*
Soft tissue profile					
UL-SnPg’ (mm)	-0.88	0.76	-0.96	1.61	0.833^€^
LL-SnPg’ (mm)	-0.42	0.89	-0.11	1.53	0.169^€^

*Statistically significant at *p*< 0.05.

The maxillary incisors had significantly greater vertical development, the maxillary molars had smaller mesial tipping and mesialization, and the mandibular molars had greater vertical development in G1 than in G2 (Table 4).

G2 presented significantly greater overjet reduction than G1. There was also significant differences in molar relationship changes because it improved toward Class I in G1 while it increased toward Class II in G2 (Table 4).

## DISCUSSION

There was good intergroup comparability regarding pre- and posttreatment ages, sex and Class II malocclusion severity. However, treatment time was significantly longer for Group 1 (Table 2). Treatment time with the Pendulum appliance was also longer than treatment with maxillary first premolar extractions in a previous study.^22^ This longer treatment time for the Jones Jig group can be attributed to several factors. Patients in this group were treated in two phases. The first phase consisted of distalization of the maxillary molars. In the second phase, a Nance button was installed for anchorage purpose, associated with fixed appliances. Leveling and alignment followed by retraction of the maxillary anterior teeth was only accomplished at this phase. However, treatment with extraction of the maxillary first premolars was usually performed in a single phase. Only some patients consisted of re-planned cases, that initially were planned for non-extraction treatment and use of extraoral headgear. However, because the patients were not using the extraoral appliance, which could cause significant anteroposterior changes and impair the comparison, it does not seem likely that this would consist in a problem in this study.

Another factor responsible for the difference in treatment times is the fact that the extraction protocol does not causes side effects. Conversely, the Jones Jig appliance causes numerous side effects, such as protrusion and labial tipping of the maxillary incisors, mesialization and mesial tipping of the maxillary premolars, which are corrected only during treatment with fixed orthodontic appliances.^2^ ^,^ ^4^ ^,^ ^7^ ^,^ ^9^ ^,^ ^10^ ^,^ ^23^ Due to lack of comparability between the treatment times of the two groups, the treatment changes ​​were “annualized” in the Jones Jig group. The method of annualization has been used in many studies, and is an effective method for a reliable comparison between groups with different treatment times.^21^ ^,^ ^24^

At pretreatment, the maxillary first premolar extractions group (G2) presented a more accentuated vertical growth pattern, associated with greater mandibular retrusion and overjet, while the Jones Jig group (G1) had more horizontal characteristics, with greater overbite (Table 3). The more accentuated vertical growth pattern of G2 may have contributed for the extraction treatment performed in these patients.^6^ ^,^ ^25^

These slight intergroup differences were expected, and their relevance is addressed throughout the discussion.

The maxillary first premolar extractions protocol produced significantly greater reduction in maxillary protrusion, and consequently greater skeletal base anteroposterior changes, than the Jones Jig appliance (Table 4). This may be consequent to the greater initial overjet of G2 and also to the amount of its correction with treatment.^23^ Besides, the maxillary incisors in Group 2 experienced greater numeric retrusion and less palatal tipping than in G1, which could have influenced greater retraction of point A. Usually, the Jones Jig appliance causes mild changes in the maxilla,^4^ ^,^ ^9^ ^,^ ^10^ and consequently in the anteroposterior apical base relationship, while greater changes are reported with maxillary first premolar extractions.^13^ ^,^ ^26^

The changes in mandibular component were similar in the groups. This similarity was expected, since both treatment protocols act essentially in the maxilla^4^ ^,^ ^9^ ^,^ ^10^ ^,^ ^12^ ^,^ ^13^ (Table 4).

Lower anterior face height experienced greater increase in the Jones Jig group (Table 4). This probably was consequent to the distalization mechanics, that usually tends to increase this variable.^2^ ^,^ ^4^ ^,^ ^9^ On the other hand, the maxillary first premolar extractions protocol seemed to provide better vertical control of lower anterior face height, allowing only a small increase, which is expected because the patients are growing.^6^ ^,^ ^14^

 The maxillary incisors in the Jones Jig group experienced extrusion, and there was intrusion in G2 (Table 4). This demonstrates that there is less vertical control of the maxillary incisors with intraoral distalizing mechanics than with maxillary first premolar extractions. This increased vertical development of the maxillary incisors in G1 may also be consequent to the greater increase in LAFH that usually accompanies distalization mechanics.^2^ ^,^ ^4^ ^,^ ^9^ On the other hand, vertical control of the maxillary incisors in G2 may have occurred with incorporation of accentuated curve of Spee in the maxillary arch.^27^ ^,^ ^28^

Behavior of the maxillary molars reflected the mechanics used in each group. The maxillary first premolar extractions group, in which the extraction spaces needed to be closed, had greater molar mesial tipping and mesialization than G1.^1^ ^,^ ^15^ Tipping occurred due to poor mechanical control of the molar, and mesialization occurred because the patients did not present complete Class II malocclusions and therefore, some mesialization was allowed.^1^ ^,^ ^15^ ^,^ ^16^ In the Jones Jig group, the small molar mesial tipping occurred during the second phase of treatment in which leveling and alignment is obtained and the anterior teeth are retracted.^10^ ^,^ ^24^ The slight mesialization in this group is consequent to the anterior maxillary displacement with growth, which overrides the amount of molar distalization, and shows mesialization in relation to PTV.^24^ ^,^ ^29^

G1 showed significantly greater mandibular molar vertical development probably due to the use of Class II elastics with fixed appliances in the second phase of treatment^30^ (Table 4).

The overjet had a greater decrease in G2 partially because this group had a greater pretreatment overjet and also because there was numerically greater retrusion of the maxillary incisors in this group. This has also been shown previously.^7^ ^,^ ^15^

The significant intergroup difference regarding molar relationship is because in non-extraction treatment it improved toward a Class I molar relationship and in the maxillary first premolar extractions group, it improved toward a more accentuated Class II relationship, because the patients did not present complete Class II malocclusions pretreatment and therefore, some molar mesialization could be allowed.^1^ ^,^ ^15^

Both protocols corrected the Class II malocclusion effectively and with the same effects on the soft tissue profile.^4^ ^,^ ^7^ ^,^ ^12^

### Clinical considerations

According to the results of this study, the main effects promoted by the treatment protocols evaluated are similar. However, some factors involved in the decision of extracting or not during orthodontic treatment are: facial profile; severity of the dental crowding; accentuated buccal inclination of the mandibular incisors; periodontal evaluation and quantity of alveolar bone; and root resorption.

Treatment of Class II malocclusion with the Jones Jig appliance is indicated for patients with a pleasant facial profile and without major skeletal compromise, since it promotes only dentoalveolar changes.^17^ However, this appliance should be avoided in patients with a vertical growth pattern.

The two treatment phases with the Jones Jig appliance, the need for patient compliance in the use of Class II elastics and time expend for correction of side effects resulting from distalization resulted in a longer treatment time with the Jones Jig appliance when compared to two maxillary first premolar extraction.

## CONCLUSIONS

» Treatment with maxillary first premolar extractions produced greater maxillary retraction, improvement in anteroposterior apical base relationship, smaller increase in lower anterior face height, greater vertical control of the maxillary incisors and greater overjet reduction.
» The two treatment protocols produced similar changes in the soft tissue profile.
» Both protocols were effective in the treatment of Class II malocclusion, but treatment time was significantly longer for Jones Jig group.
